# The Role of Objectively Measured, Altered Physical Activity Patterns for Body Mass Index Change during Inpatient Treatment in Female Patients with Anorexia Nervosa

**DOI:** 10.3390/jcm7090289

**Published:** 2018-09-18

**Authors:** Celine S. Lehmann, Tobias Hofmann, Ulf Elbelt, Matthias Rose, Christoph U. Correll, Andreas Stengel, Verena Haas

**Affiliations:** 1 Center for Internal Medicine and Dermatology, Department of Psychosomatic Medicine, Charité-Universitätsmedizin Berlin, 12200 Berlin, Germany; celine-sina.lehmann@charite.de (C.S.L.); Tobias.Hofmann@charite.de (T.H.); Ulf.Elbelt@charite.de (U.E.); Matthias.Rose@charite.de (M.R.); 2 Department of Child and Adolescent Psychiatry, Charité-Universitätsmedizin Berlin, 13353 Berlin, Germany; ccorrell@northwell.edu; 3 Center for Internal Medicine with Gastroenterology and Nephrology, Division for Endocrinology, Diabetes and Nutrition, Charité-Universitätsmedizin Berlin, 12200 Berlin, Germany; 4 Donald and Barbara Zucker School of Medicine at Hofstra/Northwell, Hempstead, NY 11549, USA; 5 Department of Psychiatry, The Zucker Hillside Hospital, Glen Oaks, NY 11004, USA; 6 Department of Psychosomatic Medicine and Psychotherapy, Medical University Hospital Tübingen, 72076 Tübingen, Germany

**Keywords:** accelerometry, eating disorders, motor restlessness, physical inactivity

## Abstract

Increased physical activity (PA) affects outcomes in patients with anorexia nervosa (AN). To objectively assess PA patterns of hospitalized AN patients in comparison with healthy, outpatient controls (HC), and to analyze the effect of PA on Body Mass Index (BMI) change in patients with AN, we measured PA in 50 female patients with AN (median age = 25 years, range = 18–52 years; mean BMI = 14.4 ± 2.0 kg/m^2^) at the initiation of inpatient treatment and in 30 female healthy controls (median age = 26 years, range = 19–53 years; mean BMI = 21.3 ± 1.7 kg/m^2^) using the SenseWear™ armband. Duration of inpatient stay and weight at discharge were abstracted from medical records. Compared with controls, AN patients spent more time in very light-intensity physical activity (VLPA) (median VLPA = 647 vs. 566 min/day, *p* = 0.004) and light-intensity physical activity (LPA) (median LPA = 126 vs. 84 min/day, *p* < 0.001) and less time in moderate-intensity physical activity (MPA) (median MPA = 82 vs. 114 min/day, *p* = 0.022) and vigorous physical activity (VPA) (median VPA = 0 vs. 16 min/day, *p* < 0.001). PA and BMI increase were not associated in a linear model, and BMI increase was mostly explained by lower admission BMI and longer inpatient stay. In a non-linear model, an influence of PA on BMI increase seemed probable (jack knife validation, *r*^2^ = 0.203; *p* < 0.001). No direct association was observed between physical inactivity and BMI increase in AN. An altered PA pattern exists in AN patients compared to controls, yet the origin and consequences thereof deserve further investigation.

## 1. Introduction

The role of increased physical activity (PA) for the onset and maintenance of anorexia nervosa (AN) is increasingly recognized. Being associated with a longer duration of inpatient treatment [1] and higher rates of a chronic outcome [2] as well as drop-out from treatment [3], increased PA can be regarded as a significant factor in the persistence of the disease [4]. However, high level PA is addressed insufficiently by current research [5]. As a consequence, a deeper understanding of the mechanisms underlying altered PA in AN as well as for the development of suitable therapeutic strategies to manage PA during weight restoration efforts are urgently warranted to improve outcomes for patients with AN.

Elevated levels of physical activity have been observed in 30–80% of patients suffering from AN [6,7], with this high range probably resulting from varying methods of PA measurement [8]. When assessed with subjective measurement tools including exercise questionnaires, patients reported higher total PA in comparison with a control group, yet simultaneous objective PA assessment using actigraphy yielded similar PA levels [9], suggesting that self-report overestimated PA in patients with AN and that objective assessments are needed to obtain accurate results. In addition, PA behavior is complex and has multiple dimensions; therefore, objective quantification of PA targets different components. Previous studies on objectively assessed PA in AN have yielded mixed results, with some reporting no differences in time spent in moderate to vigorous and daytime PA [10], or fidgeting [8], while others reported increased moderate to vigorous PA duration [3] and seated non-exercise PA [11] between AN inpatients and controls.

In a previous study [12], we focused on a potential link between high PA in AN and hypoleptinemia using a multisensor body monitor (Sensewear™ armband) for objective PA detection in hospitalized adults with AN. Results indicated that the use and interpretation of accelerometry, employed to objectively assess PA in AN patients, needs to be developed further and should also include parameters of physical inactivity. Building on the previous findings based on simple step count, the present study focused on a more detailed analysis of an expanded set of objectively measured PA patterns and intensities in adult females with AN, including inactivity parameters and adding a comparison to normal weight controls. We aimed to investigate the relationship between different PA patterns and BMI increase during inpatient treatment. We hypothesized that during inpatient treatment (I), hospitalized adult AN patients show increased low intensity PA in comparison with healthy controls, (II) increased low-level PA and BMI increase are inversely related, and (III) physical inactivity and BMI increase are directly related.

## 2. Subjects and Methods

### 2.1. Study Population

We enrolled 50 female adults with AN who were admitted to the Department of Psychosomatic Medicine at Charité—Universitätsmedizin Berlin for inpatient treatment of AN between 2011 and 2016. Patient inclusion criteria were: A diagnosis of AN according to ICD-10 (International Statistical Classification of Diseases and Related Health Problems, 10th Revision), restrictive, purging or atypical type, as well as a BMI < 17.5 kg/m^2^. Exclusion criteria were: age <18 years, current pregnancy or a diagnosed psychotic episode. Information about the duration of the illness, comorbidities as well as medication at the beginning and end of the treatment program were retrieved from anamnestic data and medical reports. Between 2015 and 2016, we also recruited 30 sex-matched and similar aged normal weight healthy controls (HC), consisting mostly of clinical staff and relatives thereof. A BMI between 18.5 and 25 kg/m^2^ served as inclusion criteria. Exclusion criteria were: Any known major medical or psychiatric disease and any condition with significant influence on PA. All participants gave written informed consent, and the study was approved by the institutional ethics committee of the Charité—Universitätsmedizin Berlin.

### 2.2. Anthropometry

Weight of all patients was measured to the nearest 0.1 kg via a digital scale (Seca 771, Vogel & Halke, Hamburg, Germany) and height to the nearest 0.5 cm via a stadiometer (Seca 220 Stadiometer, Vogel & Halke, Hamburg, Germany) [13]. Measurements took place in the morning between 7 and 8 a.m. after overnight fasting and in underwear. Weight of the controls was measured after a 2-h fast using a chair scale (MCB300K100M, KERN & Sohn GmbH, Balingen, Germany) and height was measured using a stadiometer (Vogel & Halke). BMI was calculated as kg/m^2^.

### 2.3. Bioelectrical Impedance Analysis

Whole-body bioimpedance was measured by Nutriguard-M (Data Input, Darmstadt, Germany; electrodes: Bianostic-AT, Data Input) as part of the patients’ clinical measurements. For bioimpedance analysis (BIA) of the normal-weight controls Biacorpus RX 4004 (MEDICAL HealthCare GmbH, Karlsruhe, Germany; Electrodes: BIA Classictabs, Medical HealthCare GmbH, Karlsruhe, Germany) was used. Patients and controls were weighed after fasting for at least 2 h, voiding and an equilibration period in a supine position. The equilibration period of both AN patients and controls lasted at least 10 min. BIA was carried out in accordance to the manufacturer’s instructions, and body composition was calculated with Body Comp software (Version 9.0, Professional Scientific, Medical Health Care GmbH, Karlsruhe, Germany).

### 2.4. PA Assessment

PA was measured in AN patients after inpatient admission and inclusion into the study. Using a portable armband device (SenseWear™ PRO3 armband; BodyMedia, Inc., Pittsburgh, PA, USA), PA was continuously detected over a 3-day period (Friday to Sunday). During the time of PA detection, the study population was not restricted regarding their daily physical activity [13]. A day was included into data analysis if the armband had been worn for at least 20.5 h [13]. Measurements of controls took place while they stayed in their usual environment and by using the SenseWear™ PRO3 or the SenseWear™ MF armband. According to a statement of the manufacturer from 15 March 2011, the Sensewear Pro 3 and MF models were shown to be functionally equivalent in terms of sensor technology and data analysis (manufacturers statement on equivalency available on request).

The Sensewear armband is a multi-sensor device worn on the upper dominant arm which enables a continuous physiological PA detection [14] by measuring parameters such as heat flow, galvanic skin response (GSR), body temperature and near-body temperature [15]. An integrated two-axial accelerometer captures the movement of the upper arm as well as the position of the body [14]. The information captured by the five sensors and participant characteristics (age, sex, weight, height, smoker or non-smoker and handedness) [16] are integrated and analyzed by a proprietary software (SenseWear™ Software, Version 8.0, BodyMedia, Inc., Pittsburgh, PA, USA). This program is based upon algorithms of the manufacturer and able to analyze the collected raw data at different metabolic equivalent (MET) values. The latter represents a standardized indicator which is independent of time, body weight and sex [15]. One MET is equivalent to 1 kcal/h/kg body weight and serves as useful parameter to describe the energy expenditure [17] and intensity [15] of a specific activity. The MET value ranges from 1 MET while at rest [17] and 1.1 METs when driving in a car to 2–4 METs when doing housework [15], and can reach maximum values of 20 METs when doing excessive sports [15]. According to previous studies, we used six different MET categories to classify different activity intensities of PA within our AN and control group:

- A MET-value ≤1.0 was defined as the rate of energy expenditure while at rest [17].
- Activities with a MET-value ≤1.8 were considered as sedentary behavior [18].
- Thus, we concluded to form a new category ranging from ≥1.1 to ≤1.8 METs to describe very light-intensity physical activities (VLPA).
- Light-intensity physical activities (LPA) were defined as MET-values >1.8 and <3 [18].
- Moderate-intensity activities (MPA) were defined as ≥3 METs to <6 METs [17,18].
- Vigorous-intensity activities (VPA) were divided firstly into MET-values ranging from ≥6 to ≤9, and secondly into values >9 METs [3].

### 2.5. Statistical Analysis

Based on a prior study of 11 AN patients and 10 HCs whose activity was measured with a shoe-based accelerometer at three time points: (I) while eating lunch, (II) filling out questionnaires, and (III) watching television for 1 h, power was sufficient with 19 analyzed individuals to demonstrate a significant difference in total PA levels (df = 1.19, f = 5.68, *p* = 0.03) [11]. However, we aimed to assess activity continuously for 3 days and parse the analyses into six different PA intensity levels, i.e., (I) at rest, (II) very light, (III) light, (IV) moderate, (V) vigorous and (VI) vigorous >9 METs. Therefore, we assumed that at least four times more patients (i.e., *n* = 44) would be required to have sufficient power. For organizational purposes we capped HCs at *n* = 30 (assuming less heterogeneity among HCs); we increased the sample size of AN patients to *n* = 50.

A *p*-value of <0.05 was set as the significance threshold. All variables were tested in a two-sided fashion. All data are presented as mean ± standard deviation (SD) if following a normal distribution, otherwise as median (25th/75th percentile), or absolute frequency (relative frequency %). Quartiles were computed using R type 8 so that the resulting quantile estimates were approximately median-unbiased, regardless of the distribution. Data following a Gaussian distribution were analyzed by *t*-tests. Wilcoxon tests were applied for group differences for quantitative response variables not following a Gaussian distribution. Analyses for categories were performed by Fisher’s exact test. To test the relationship between BMI change and various potential predictors, univariate and multivariate linear models were computed. A regression tree was computed, as this approach does not make assumptions on distributions or linearity. This machine learning technique computes a series of prediction thresholds to split a data set. Given our relatively small sample, splitting the data set into learning and test sets was not feasible; therefore, we applied a jack-knife procedure, classifying each subject based on a tree build from the remaining patients. Statistical analyses were computed using R version 3.4.2, R Core Team 2017.

## 3. Results

### 3.1. Characterization of the Study Population Including Medical Details, Comorbidities, Medications, and Body Composition

Table 1 shows the patients’ demographic characteristics and body composition data upon hospital admission compared to the healthy control group. The two study groups did not significantly differ in age (*p* = 0.057). Body weight, BMI, body fat, and lean mass were significantly lower in patients with AN compared to controls (all: *p* < 0.001; Table 1). Regarding phase angle, i.e., the ratio of body cell mass to fat-free mass as an indicator of cellular health and integrity, AN patients had significantly lower values than controls (*p* < 0.001; Table 1).

Medical details, comorbidities, and current medications of the study populations are summarized in a supplemental table (Supplementary Table S1). Forty-eight percent of the patients were diagnosed with restrictive AN, 26% with purging AN, and 26% with atypical AN. In terms of comorbidities, AN patients had significantly more pericardial effusion (*p* < 0.001), episodes of depression (*p* < 0.001), and at least one comorbidity (AN = 96% vs. C = 33%, *p* < 0.001). No statistically significant differences existed for other medical disorders. In terms of medication, a significant difference between both groups existed only for psychopharmacological treatment, with none of the controls (C) but 16% of the AN patients receiving medication on admission (*p* = 0.021). No significant difference existed for oral contraceptives (*p* = 0.052), L-thyroxine (*p* = 0.632), or taking no medication (AN, 35% vs. C, 37%; *p* = 1.000).

### 3.2. Comparison of Physical Activity and MET Intensities

PA data and time spent in different levels of physical activity of 50 hospitalized AN patients compared to 30 ambulatory healthy controls are outlined in Table 2. Both groups engaged in similar levels of activity in terms of average steps and total distance per day. However, patients with AN had a greater range regarding the step count; 2479–31876 vs. 6507–22948 steps (Table 2). Significant differences were observed in daily average METs with patients presenting lower median values than controls. Patients with AN spent significantly more time in very low (*p* = 0.004) and low (*p* < 0.001) levels of PA than controls. Conversely, AN patients spent significantly less time in PA below the very low PA level (*p* < 0.001), in moderate (*p* = 0.022) as well as in 6–9 MET vigorous activity level (*p* < 0.001; Table 2). However, no significant differences were found for markers of physical inactivity: Both groups spent nearly the same duration of time on recumbency and sleep.

### 3.3. BMI Change

Table 3 summarizes clinical outcome parameters of AN patients on admission and at discharge from inpatient treatment. On average, AN patients achieved a weight gain of 2.1 ± 2.3 kg during the 32-day (25th percentile: 26 days; 75th percentile: 63 days) inpatient treatment program. The BMI increased by 0.7 ± 0.8 kg/m^2^, which is equivalent to a BMI increase of 4%. The mean rate of weight gain in AN was 0.29 kg/week and ranged from −0.44 kg/week up to 1.35 kg/week. Seven (14%) out of the 50 AN patients lost weight during their inpatient stay.

### 3.4. Association between Physical Activity and Clinical Outcome

**Figure 1:**
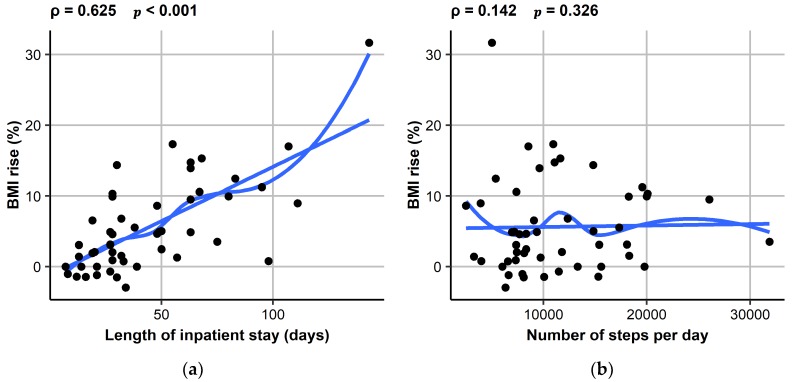
Associations between BMI increase in % and (**a**) length of inpatient stay and (**b**) number of steps per day applying Spearman rank correlation. BMI, Body Mass Index.

In an univariate regression analysis with BMI increase in % as the dependent variable and a range of potential predictive factors as independent variables (length of inpatient stay, phase angle, BMI on admission, steps, total distance, PA at different MET intensities, duration of sleep and recumbency), only length of inpatient stay (*r* = 0.154; *p* < 0.001), phase angle (*r* = −2.95; *p* = 0.002) and BMI on admission (*r* = −1.99; *p* < 0.001) were significant predictors (presented in Figure 1 with Spearman rank correlation). In a multivariable model, length of inpatient stay (*p* < 0.001) and BMI on admission (*p* = 0.029) remained significant predictors and duration of sleep became significant (*r* = −0.0107; *p* = 0.019) as well. In addition, for MPA a trend (*r* = 0.0111; *p* = 0.089) towards becoming a significant positive predictor of BMI increase was observed.

**Figure 2:**
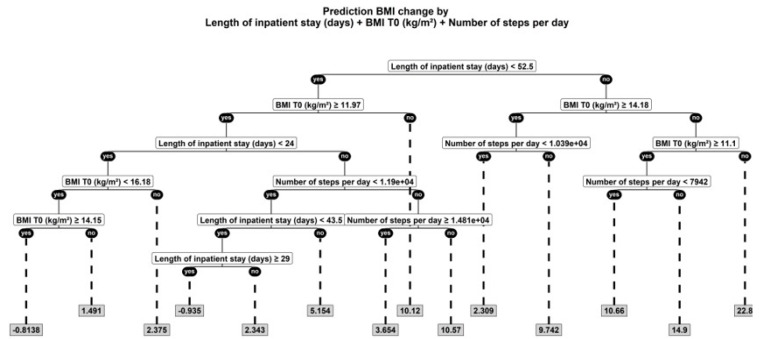
Regression tree for non-linear modelling to test the relation between BMI percent change and further parameters. BMI, Body Mass Index.

In an exploratory regression tree model (Figure 2), the following parameters were relevant predictors of percent BMI change: length of inpatient stay, BMI on admission, and number of steps.

**Figure 3:**
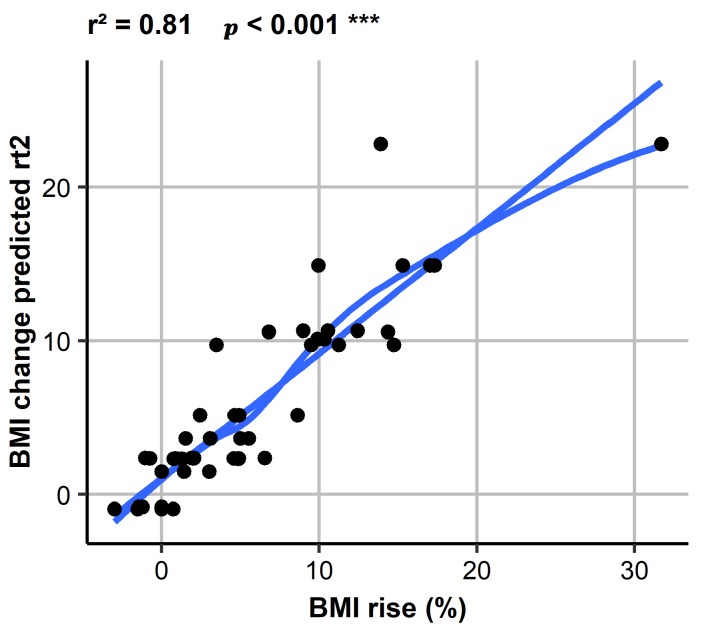
Non-linear model on predicted vs. measured BMI change. BMI, Body Mass Index.

With this non-linear model, the association between actual and predicted BMI percent change could be predicted with an *r*^2^ = 0.81 (Figure 3).

Furthermore, validation of this prediction model by jack-knife analysis was successful (*r*^2^ = 0.203; *p* < 0.001). The importance score for length of inpatient stay was 1026, for admission BMI 822, and for number of steps 453, potentially suggesting at least a small effect of PA measured as steps on % BMI increase. Applying these statistical procedures also for LPA as a parameter for low level activity, data yielded a similar value for *r*^2^ for steps as well as an importance score of 562 for LPA, indicating also a slight effect of LPA on BMI change comparable in strength to that of steps.

## 4. Discussion

In our study, the following main results emerged: (1) Compared with healthy controls, AN patients spent more time engaging in light and less time engaging in vigorous intensity PA; (2) the patient’s BMI increase during inpatient treatment was largely predicted by low BMI on admission and longer duration of inpatient stay; (3) high step count and time in light-intensity PA only emerged as potential predictors of lower BMI increase in an exploratory and non-linear model; and (4) contrary to our assumption, the duration of sleep as a marker of physical inactivity was inversely associated with BMI increase.

Few studies have objectively measured low intensity PA in AN inpatients compared with healthy controls. Our findings demonstrating increased low intensity PA are consistent with a previous study using a shoe-based monitor [11]. Using the SenseWear armband, El Ghoch et al. [3] also observed that AN patients spent less time in high intensity PA, yet contrary to our findings time spent in light-intensity PA did not differ between groups and the patients showed a significantly higher moderate and vigorous PA duration. The division into two low intensity MET categories (1.1–1.8 and 1.8–3) in the present study as opposed to one category (1.1–3 METs) in the study by El Ghoch [3] may explain the different and more detailed results. Differences in time spent with moderate to vigorous PA might relate to varying approaches with respect to the restriction of PA on the wards or to practical opportunities to exercise in the environment outside the ward. The choice of different PA assessment tools should also be taken into account: When assessing PA with movement sensors, there was no difference in time spent on “fidgeting” (operationalized as “body position change counts”) between AN patients and controls [8]. Yet, the authors of that study mentioned problems with measurement technology consisting of several leads and wires, which might have affected compliance and PA behavior of the study participants.

A better understanding of the origin of distinct PA patterns in AN patients is warranted. Increased light PA in AN might be a consequence of negative energy balance resulting in a foraging response to increase PA to find food [19,20] or linked with a distinct phenotype characterized by disturbed energy homeostasis specifically associated with increased PA despite severe weight loss [6,21]. Further origins for increased light PA in AN might be an attempt of emotion regulation [6,22] or the desire to lose weight [4,23]. Interestingly, when interviewed 57 years after participating in the Minnesota starvation experiment in 1944/1945, the volunteer men did not report an increased drive for PA while starving [23]. To add to the complexity of altered PA in AN, the surrounding conditions during the time of PA assessment might play a considerable role. When obese volunteers were subjected to 24-h measurements of energy turnover within a metabolic chamber [24] for analysis of spontaneous PA, with exercise being prohibited within the chamber, the authors hypothesized that such forced reduction of voluntary exercise may have resulted in the partially observed increased engagement in spontaneous PA [25]. Similarly, in 16 healthy, male volunteers who underwent 8 weeks of experimental overfeeding, two-thirds of the increase in total daily energy expenditure was due to increased non-exercise activity thermogenesis (NEAT) [26]. Individual variation in NEAT accounted for the 10-fold differences in fat storage that occurred with overfeeding, suggesting that during positive energy balance, high activation of NEAT results in difficulties to gain weight for some individuals. The phenomenon of high NEAT and concomitantly energy needs of 4000 kcal/day to gain weight was recently documented in a case report of a young woman with AN at the end of therapy [27]. We believe that it is important for the tailoring of suitable PA interventions for AN patients to find an answer to the question whether increased low-level PA is an AN-specific phenotype that is linked with physiological processes during starvation and refeeding, or whether such PA behavior is related to restrictive treatment setting characteristics irrespective of AN, which may also be observable in other populations. Therefore, the current restrictive handling of PA during AN treatment may need to be reconsidered since an increase in low-level PA could provoke higher daily energy expenditure and might hinder weight recovery. Increased voluntary exercise could be accompanied by a decrease in spontaneous PA [25]. Concomitantly, Calogero et al. [28] investigated the effectiveness of an exercise program in patients with eating disorders, reporting on weight improvements through this intervention and concluding that patients in the exercise program may have been less likely to exercise secretly, whereas patients in the control group may have exercised unsupervised.

A low admission BMI was identified as a major determinant for BMI increase in AN. Resting energy expenditure proportionally declines with BMI [29], physiologically leading to a more rapid weight regain at the beginning of treatment. Longer inpatient stay also predicted BMI increase which may be at least in part explained by the rules in our adult treatment setting where patients were discharged if they continuously failed to meet the expected weight targets. On the other hand, patients who stayed in treatment longer also had more time to gain weight. Since only a slight effect of PA on BMI increase was observed and only by conducting an exploratory analysis, the admission BMI and duration of stay had an overall much greater, independent and overriding predictive power on BMI increase in AN. Whether an association between PA and weight trajectory in AN can be detected may depend on certain study characteristics, i.e., measurement technique and time point of PA assessment. There was no association between (I) PA duration at different MET intensities and daily steps at inpatient discharge and BMI at 1-year follow up [30]; (II) PA level operationalized as the average acceleration in m/s^2^/min from both feet and BMI or rate of weight gain in AN patients admitted to an inpatient unit [11], and (III) time spent on feet at low-weight within 2 weeks of hospital admission or 1-month post-treatment discharge and 12 months BMI trajectory [8]. However, a longer on-feet duration at the inpatient weight restored time point was associated with a more rapid decrease in BMI over the 12 months following discharge [8]. Interestingly, a retrospective study applying questionnaires for PA assessment 6 months and 1 week prior to inpatient admission in 20 adolescents with AN found that an increase in PA—and not a decrease in food intake—was associated with the need for inpatient treatment [31]. These findings give rise to at least some effect of PA on the weight and illness trajectory of AN, and stress the need for further, systematic studies on this topic.

In the present study, no linear and direct associations between sleep duration and recumbency, conceptualized as physical inactivity parameters, and weight gain during AN treatment existed. Similarly, others could not find associations between sleep patterns and BMI [32], or between changes in sleep patterns and changes in BMI [33]. In the latter study, there was a significant direct association between baseline sleep time and BMI. In the present study, the contribution of sleep duration to variance of BMI increase was only of minor effect size. However, unexpectedly, in a multivariable model, the duration of sleep was inversely associated with BMI increase. In obesity, short sleep duration is known to be associated with increased food intake and excess body weight [34,35]. Whether this link also applies to patients with AN needs further investigation.

While we used objective PA assessments at standardized time points close to hospital admission, which are different from other studies that assessed PA across various stages of AN treatment [11], our findings also need to be interpreted within their limitations. Firstly, the validity of the SenseWear armband in severely underweight AN patients is unknown, and raw data and algorithms within the armband software are not accessible to researchers. Nonetheless, we consider this technology suitable for PA detection due to its easy handling compared to other devices [8] and the fact that multiple sensors enable the distinction between various types of PA, the recording of actual on-body time as well as time spent on sleep [14]. Second, whether our controls were of comparable socioeconomic background, and whether the wearing of the armband motivated them to work out more than usual remains unclear. Given that PA analysis was conducted between hospitalized AN patients and healthy controls within their everyday environment and thus in two very different settings, comparability of data may be argued. As a consequence, PA patterns of the patients in the present study may not be representative of other patients with AN under other types of care. However, finding a suitable control group for hospitalized patients is difficult, as healthy people are not hospitalized, and hospitalized patients for other reasons than AN are likely to suffer from a medical condition which affects PA patterns.

In conclusion, we found that AN patients spent more time engaging in light, and less time engaging in vigorous intensity PA than controls, and that the BMI increase during inpatient treatment was predicted by low admission BMI and longer inpatient treatment. Furthermore, high step count and time in light-intensity PA only emerged as potential predictors of lower BMI increase in an exploratory and non-linear model. This latter finding indicates that the effect of PA on the disease course of AN should be quantified and clarified further and that more complex models may need to be employed in future research on this topic. Since PA behavior is likely influenced by multiple factors including age, psychological and nutritional parameters, assessment of these potential modifiers in future studies may contribute to a better understanding of PA variability in AN.

## Figures and Tables

**Table 1:** Demographic characteristics and bioimpedance data in patients with anorexia nervosa on admission and in the healthy control group.

Measurement Parameters	Anorexia Nervosa Baseline (*n* = 50)	Controls (*n* = 30)	*p*
Demographic parameters			
Age (years)	25 (21/30)	26 (23/35)	0.057
Weight (kg)	39.9 ± 6.6 (28.4–58.8)	60.5 ± 5.8 (51.2–71.9)	<0.001
Height (cm)	166 ± 7 (152–185)	169 ± 6 (159–180)	0.128
BMI (kg/m^2^)	14.4 ± 2.0 (8.9–17.7)	21.3 ± 1.7 (18.8–25.0)	<0.001
Duration of illness (months)	72 (15/134)	N/A	
Body composition			
Phase angle (°)	4.5 (3.8/5.1)	5.9 (5.5/6.4)	<0.001
Fat mass (kg)	2.9 ± 2.7 (1–12.5)	16.0 ± 3.1 (11.3–22.5)	<0.001
Fat mass (%)	6.7 ± 5.2 (2.1–21.6)	26 ± 3 (21–32)	<0.001
Fat-free mass (kg)	37 ± 4 (27–46)	44 ± 3 (39–53)	<0.001
Fat-free mass (%)	93 ± 5 (78–98)	74 ± 3 (68–79)	<0.001

Data are expressed as mean ± SD (range) or as median (25th/75th percentile). BMI, Body Mass Index; N/A, not applicable; AN, anorexia nervosa.

**Table 2:** Physical activity and the division into different MET cut-offs in patients with anorexia nervosa on admission and in the healthy control group.

Measurement Parameters	Anorexia Nervosa Baseline (*n* = 50)	Controls (*n* = 30)	*p*
Physical activity			
Number of steps per day	11,305 ± 6064 (2479–31,876)	11,098 ± 3973 (6507–22,948)	0.854
Total distance (km/day)	10.2 ± 5.5 (2.3–25.2)	9.8 ± 4.0 (4.6–19.2)	0.769
Metabolic equivalents (METs per day)	1.40 (1.40/1.60)	1.70 (1.50/1.80)	<0.001
Duration of recumbency (min/day)	483 (443/527)	500 (440/560)	0.348
Duration of sleep (min/day)	427 (375/457)	408 (363/484)	0.842
PA ≤ 1 METs duration (min/day)	496 (448/536)	588 (502/643)	<0.001
VLPA 1.1–1.8 METs duration (min/day)	647 (569/703)	566 (499/631)	0.004
LPA 1.8–3 METs duration (min/day)	126 (92/188)	84 (71/108)	<0.001
MPA 3–6 METs duration (min/day)	82 (44/130)	114 (79/165)	0.022
VPA 6–9 METs duration (min/day)	0 (0/3)	16 (8/35)	<0.001
VPA > 9 METs duration (min/day)	0.0 (0.0/0.0)	0.0 (0.0/3.2)	0.063

Data are expressed as mean ± SD (range) or as median (25th/75th percentile). LPA, light-intensity physical activity; MET, metabolic equivalent; MPA, moderate-intensity physical activity; PA, physical activity; VLPA, very light-intensity physical activity; VPA, vigorous-intensity physical activity.

**Table 3:** Clinical outcome parameters of patients with anorexia nervosa.

Measurement Parameters	Anorexia Nervosa Baseline (*n* = 50)	Anorexia Nervosa Discharge (*n* = 50)	*p*
Clinical outcome			
Weight (kg)	39.9 ± 6.6 (28.4–58.8)	42.0 ± 6.2 (31.4–59.7)	<0.001
Total weight gain (kg)	-	2.1 ± 2.3 (−1.4–9.6)	
BMI (kg/m^2^)	14.4 ± 2.0 (8.9–17.7)	15.2 ± 1.8 (11.7–18.3)	<0.001
BMI increase (kg/ m^2^)	-	0.7 ± 0.8 (−0.5–2.8)	
BMI increase (%)	-	4 (1/10)	

Data are expressed as mean ± SD (range) or as median (25th/75th percentile); BMI, Body Mass Index.

## Supplementary Materials

The following are available online at http://www.mdpi.com/2077-0383/7/9/289/s1, Table S1: Medical details, comorbidities, and medication of study patients and the healthy controls.

## References

[1] Solenberger S.E. (2001). Exercise and eating disorders: A 3-year inpatient hospital record analysis. Eat. Behav..

[2] Strober M., Freeman R., Morrell W. (1997). The long-term course of severe anorexia nervosa in adolescents: Survival analysis of recovery, relapse, and outcome predictors over 10–15 years in a prospective study. Int. J. Eat. Disord..

[3] El Ghoch M., Calugi S., Pellegrini M., Milanese C., Busacchi M., Battistini N.C., Bernabè J., Dalle Grave R. (2013). Measured physical activity in anorexia nervosa: Features and treatment outcome. Int. J. Eat. Disord..

[4] Carrera O., Adan R.A., Gutierrez E., Danner U.N., Hoek H.W., van Elburg A.A., Kas M.J. (2012). Hyperactivity in anorexia nervosa: Warming up not just burning-off calories. PLoS ONE.

[5] Gümmer R., Giel K.E., Schag K., Resmark G., Junne F.P., Becker S., Zipfel S., Teufel M. (2015). High levels of physical activity in anorexia nervosa: A systematic review. Eur. Eat. Disord. Rev..

[6] Kostrzewa E., van Elburg A.A., Sanders N., Sternheim L., Adan R.A., Kas M.J. (2013). Longitudinal changes in the physical activity of adolescents with anorexia nervosa and their influence on body composition and leptin serum levels after recovery. PLoS ONE.

[7] Rizk M., Lalanne C., Berthoz S., Kern L., Godart N., EVHAN Group (2015). Problematic exercise in anorexia nervosa: Testing potential risk factors against different definitions. PLoS ONE.

[8] Gianini L.M., Klein D.A., Call C., Walsh B.T., Wang Y., Wu P., Attia E. (2016). Physical activity and post-treatment weight trajectory in anorexia nervosa. Int. J. Eat. Disord..

[9] Keyes A., Woerwag-Mehta S., Bartholdy S., Koskina A., Middleton B., Connan F., Webster P., Schmidt U., Campbell I.C. (2015). Physical activity and the drive to exercise in anorexia nervosa. Int. J. Eat. Disord..

[10] Sauchelli S., Arcelus J., Sánchez I., Riesco N., Jiménez-Murcia S., Granero R., Gunnard K., Baños R., Botella C., de la Torre R. (2015). Physical activity in anorexia nervosa: How relevant is it to therapy response?. Eur. Psychiatry.

[11] Belak L., Gianini L., Klein D.A., Sazonov E., Keegan K., Neustadt E., Walsh B.T., Attia E. (2017). Measurement of fidgeting in patients with anorexia nervosa using a novel shoe-based monitor. Eat. Behav..

[12] Stengel A., Haas V., Elbelt U., Correll C.U., Rose M., Hofmann T. (2017). Leptin and physical activity in adult patients with anorexia nervosa: Failure to demonstrate a simple linear association. Nutrients.

[13] Hofmann T., Elbelt U., Ahnis A., Kobelt P., Rose M., Stengel A. (2014). Irisin levels are not affected by physical activity in patients with anorexia nervosa. Front. Endocrinol. (Lausanne).

[14] Andre D., Pelletier R., Farringdon J., Safier S., Talbott W., Stone R., Vyas N., Trimble J., Wolf D., Vishnubhatla S. (2006). The Development of the SenseWear® Armband, a Revolutionary Energy Assessment Device to Assess Physical Activity and Lifestyle. http://1fw.dotfit.com/sites/63/templates/categories/images/1783/Dev_SenseWear_article.pdf.

[15] Das Armband Kompendium. http://www.body-coaches.de/wp-content/uploads/Armband_Anleitung.pdf.

[16] Gastin P.B., Cayzer C., Dwyer D., Robertson S. (2018). Validity of the ActiGraph GT3X+ and BodyMedia SenseWear Armband to estimate energy expenditure during physical activity and sport. J. Sci. Med. Sport.

[17] Physical Activity Guidelines Advisory Committee (2008). Physical Activity Guidelines for Americans.

[18] Scheers T., Philippaerts R., Lefevre J. (2012). Patterns of physical activity and sedentary behavior in normal-weight, overweight and obese adults, as measured with a portable armband device and an electronic diary. Clin. Nutr..

[19] Sternheim L., Danner U., Adan R., van Elburg A. (2015). Drive for activity in patients with anorexia nervosa. Int. J. Eat. Disord..

[20] Adan R.A., Hillebrand J.J., Danner U.N., Cardona Cano S., Kas M.J., Verhagen L.A. (2011). Neurobiology driving hyperactivity in activity-based anorexia. Curr. Top. Behav. Neurosci..

[21] Casper R.C. (2016). Restless activation and drive for activity in anorexia nervosa may reflect a disorder of energy homeostasis. Int. J. Eat. Disord..

[22] Bratland-Sanda S., Sundgot-Borgen J., Rø Ø., Rosenvinge J.H., Hoffart A., Martinsen E.W. (2010). Physical activity and exercise dependence during inpatient treatment of longstanding eating disorders: An exploratory study of excessive and non-excessive exercisers. Int. J. Eat. Disord..

[23] Eckert E.D., Gottesman I.I., Swigart S.E., Casper R.C. (2018). A 57-year follow-up investigation and review of the Minnesota study on human starvation and its relevance to eating disorders. Arch. Psychol..

[24] Ravussin E., Lillioja S., Anderson T.E., Christin L., Bogardus C. (1986). Determinants of 24-hour energy expenditure in man. Methods and results using a respiratory chamber. J. Clin. Investig..

[25] Garland T., Schutz H., Chappell M.A., Keeney B.K., Meek T.H., Copes L.E., Acosta W., Drenowatz C., Maciel R.C., van Dijk G. (2011). The biological control of voluntary exercise, spontaneous physical activity and daily energy expenditure in relation to obesity: Human and rodent perspectives. J. Exp. Biol..

[26] Levine J.A., Eberhardt N.L., Jensen M.D. (1999). Role of nonexercise activity thermogenesis in resistance to fat gain in humans. Science.

[27] Haas V., Stengel A., Mähler A., Gerlach G., Lehmann C., Boschmann M., de Zwaan M., Herpertz S. (2018). Metabolic barriers to weight gain in patients with anorexia nervosa: A young adult case report. Front. Psychiatry.

[28] Calogero R.M., Pedrotty K.N. (2004). The practice and process of healthy exercise: An investigation of the treatment of exercise abuse in women with eating disorders. Eat. Disord..

[29] Haas V.K., Gaskin K.J., Kohn M.R., Clarke S.D., Müller M.J. (2010). Different thermic effects of leptin in adolescent females with varying body fat content. Clin. Nutr..

[30] El Ghoch M., Calugi S., Pellegrini M., Chignola E., Dalle Grave R. (2016). Physical activity, body weight, and resumption of menses in anorexia nervosa. Psychiatry Res..

[31] Higgins J., Hagman J., Pan Z., MacLean P. (2013). Increased physical activity not decreased energy intake is associated with inpatient medical treatment for anorexia nervosa in adolescent females. PLoS ONE.

[32] Delvenne V., Kerkhofs M., Appelboom-Fondu J., Lucas F., Mendlewicz J. (1992). Sleep polygraphic variables in anorexia nervosa and depression: A comparative study in adolescents. J. Affect. Disord..

[33] El Ghoch M., Calugi S., Bernabè J., Pellegrini M., Milanese C., Chignola E., Dalle Grave R. (2016). Sleep patterns before and after weight restoration in females with anorexia nervosa: A longitudinal controlled study. Eur. Eat. Disord. Rev..

[34] Wu Y., Zhai L., Zhang D. (2014). Sleep duration and obesity among adults: A meta-analysis of prospective studies. Sleep Med..

[35] Cappuccio F.P., Taggart F.M., Kandala N.B., Currie A., Peile E., Stranges S., Miller M.A. (2008). Meta-analysis of short sleep duration and obesity in children and adults. Sleep.

