# Supplementary material for: The Role of Objectively Measured, Altered Physical Activity Patterns for Body Mass Index Change during Inpatient Treatment in Female Patients with Anorexia Nervosa
Source: J Clin Med. 2018 Sep 18;7(9):289. doi: 10.3390/jcm7090289 (PMC6162412; doi:10.3390/jcm7090289)
Supplement: Supplementary file 1 [file jcm-07-00289-s001.pdf]

Table S1. Medical details, comorbidities, and medication of study patients and the healthy controls.

	Anorexia nervosa (n=50)	Controls (n=30)	p
**Medical details**			
**Length of inpatient stay** (days)	32 (26 / 63)	NA	
**Anorexia nervosa subtype**			
restrictive	24 (48%)	NA	
purging	13 (26%)	NA	
atypical	13 (26%)	NA	
**Comorbidities**			
Missing data	0 (0%)	6 (20%)	
Pericardial effusion	13 (26%)	0 (0%)	<0.001
Episode of depression (moderately)	13 (26%)	0 (0%)	<0.001
Ascites	6 (12%)	0 (0%)	0.079
Selfharming behaviour	3 (6%)	0 (0%)	0.288
Osteopenia	5 (10%)	0 (0%)	0.151
Iron deficiency anaemia	1 (2%)	1 (3%)	1.000
Hashimoto's disease	1 (2%)	1 (3%)	1.000
Atopic dermatitis	1 (2%)	1 (3%)	1.000
Hip dysplasia	0 (0%)	1 (3%)	0.375
No comorbidities	2 (4%)	20 (67%)	<0.001
**Medication on admission**			
Missing data	1 (2%)	0 (0%)	
Oral contraceptives	13 (27%)	15 (50%)	0.052
Other contraceptives	0 (0%)	2 (7%)	0.141
Protein energy drink	1 (2%)	0 (0%)	1.000
Iron products	3 (6%)	0 (0%)	0.284
Psychopharmacological treatment	8 (16%)	0 (0%)	0.021
L-thyroxine	2 (4%)	2 (7%)	0.632
No medication	17 (35%)	11 (37%)	1.000
**Medication at discharge**			
Missing data	3 (6%)	NA	
Oral contraceptives	12 (26%)	NA	
Other contraceptives	0 (0%)	NA	
Protein energy drink	16 (34%)	NA	
Iron products	4 (9%)	NA	
Psychopharmacological treatment	18 (38%)	NA	
L-thyroxine	0 (0%)	NA	
No medication	6 (13%)	NA	

Data are expressed as mean ± SD (range) or absolute frequency (relative frequency %). NA, not applicable.
